# Transcriptional and Proteomic Choreography Under Phosphorus Deficiency and Re-supply in the N_2_ Fixing Cyanobacterium *Trichodesmium erythraeum*

**DOI:** 10.3389/fmicb.2019.00330

**Published:** 2019-03-05

**Authors:** Kyle R. Frischkorn, Sheean T. Haley, Sonya T. Dyhrman

**Affiliations:** ^1^Department of Earth and Environmental Sciences, Columbia University, New York, NY, United States; ^2^Lamont-Doherty Earth Observatory, Palisades, NY, United States

**Keywords:** diazotroph, transcriptome (RNA-seq), proteome, phosphorus (deficiency, uptake), biological oceanography, phosphorus metabolism, cyanobacterium, *Trichodesmium*

## Abstract

The N_2_ fixing cyanobacterium *Trichodesmium* is a critically important organism in oligotrophic marine ecosystems, supplying “new” nitrogen (N) to the otherwise N-poor tropical and subtropical regions where it occurs. Low concentrations of phosphorus (P) in these regions can constrain *Trichodesmium* distribution and N_2_ fixation rates. Physiological characterization of a single species in a mixed community can be challenging, and ‘omic approaches are increasingly important tools for tracking nutritional physiology in a taxon-specific manner. As such, studies examining the dynamics of gene and protein markers of physiology (e.g., nutrient stress) are critical for the application and interpretation of such ‘omic data *in situ*. Here we leveraged combined transcriptomics, proteomics, and enzyme activity assays to track the physiological response of *Trichodesmium erythraeum* IMS101 to P deficiency and subsequent P re-supply over 72 h of sampling. P deficiency resulted in differential gene expression, protein abundance, and enzyme activity that highlighted a synchronous shift in P physiology with increases in the transcripts and corresponding proteins for hydrolyzing organic phosphorus, taking up phosphate with higher affinity, and modulating intracellular P demand. After P deficiency was alleviated, gene expression of these biomarkers was reduced to replete levels within 4 h of P amendment. A number of these gene biomarkers were adjacent to putative pho boxes and their expression patterns were similar to a *sphR* response regulator. Protein products of the P deficiency biomarkers were slow to decline, with 84% of the original P deficient protein set still significantly differentially expressed after 72 h. Alkaline phosphatase activity tracked with proteins for this enzyme. With the rapid turnover time of transcripts, they appear to be good biomarkers of a P stress phenotype, whereas proteins, with a slower turnover time, may better reflect cellular activities. These results highlight the importance of validating and pairing transcriptome and proteome data that can be applied to physiological studies of key species *in situ*.

## Introduction

*Trichodesmium* is a keystone member of marine microbial communities in the oligotrophic tropical and subtropical oceans: the dinitrogen (N_2_) it fixes is estimated to contribute half of the total biologically fixed nitrogen (N) in marine systems, a limiting resource that fuels primary productivity and the subsequent sequestration of carbon from the atmosphere to the deep ocean (Capone et al., 1997; Mahaffey et al., 2005; Bergman et al., 2013). *Trichodesmium* physiological ecology and N_2_ fixation can be limited by resources like phosphorus (P) and iron (Fe) (Sañudo-Wilhelmy et al., 2001; Sohm et al., 2011; Chappell et al., 2012; Moore et al., 2013; Jiang et al., 2018; Polyviou et al., 2018; Rouco et al., 2018). Dissolved inorganic phosphate (DIP) is vanishingly low across the oligotrophic oceans (Paytan and McLaughlin, 2007; Karl, 2014) and subject to rapid turnover rates, particularly in warm surface waters where *Trichodesmium* is found (Moutin et al., 2005; Karl, 2014). For example, there have been an increasing number of studies that suggest *Trichodesmium* populations are P-stressed in the oligotrophic North Atlantic Subtropical Gyre (NASG) where surface DIP can be subnanomolar (Wu et al., 2000; Dyhrman et al., 2002; Mills et al., 2004; Webb et al., 2007; Moore et al., 2009), and may limit growth and N_2_ fixation. Herein, P stress describes an inducible and multifaceted physiological response specifically to low P (*sensu* Dyhrman and Ruttenberg, 2006; Dyhrman, 2016). For example, in low P conditions the induction of typical P stress responses, e.g., high affinity P transporters or alkaline phosphatases (APs), will result in either alleviation of stress or limitation of *Trichodesmium* growth and N_2_ fixation. Patterns of P stress can indicate where P supply is a driver of *Trichodesmium* physiological ecology.

Tracking the P physiology of *Trichodesmium in situ* is critical for understanding constraints on N_2_ fixation and *Trichodesmium* biogeography, but is challenging in several regards. *Trichodesmium* lives with a diverse consortium of epibiotic bacteria (Nausch, 1996; Hmelo et al., 2012; Frischkorn et al., 2017; Lee et al., 2017) that are known to manipulate P cycling within the colony (Van Mooy et al., 2012). The presence of such a complex microbiome can drive variation in apparent uptake and enzyme hydrolysis rates, making these common measures of P physiology not necessarily *Trichodesmium*-specific. Further, *Trichodesmium* is known to modulate its P quota (Bertilsson et al., 2003; White et al., 2006; Van Mooy et al., 2009), and use alternative P forms like phosphite (Polyviou et al., 2015) and dissolved organic phosphorus (DOP) (Mulholland et al., 2002; Dyhrman et al., 2006 and references therein), in order to meet P demand. The bioavailability and flux of phosphite and DOP to *Trichodesmium* is not directly quantifiable, leaving uncertainties as to the extent to which P bioavailability constrains *Trichodesmium* growth and N_2_ fixation. Taken together, these challenges have driven work to develop and apply biomarkers of P stress and to track cellular activities using ‘omics approaches which can be applied in a species-specific manner.

Metatranscriptome and metaproteome approaches are increasingly used to track phytoplankton physiological ecology (Sowell et al., 2009; Dong et al., 2014; Ottesen et al., 2014; Aylward et al., 2015; Wilson et al., 2017; Frischkorn et al., 2018), where gene expression and protein abundance may be used to assay for a given phenotype (e.g., P stress), or screen for putative activities (e.g., phosphonate use). Given the importance of these field approaches, there are an increasing number of transcriptome and proteome studies which provide critical baseline information on how transcript and protein biomarkers are modulated with Fe, P, or Fe-P co-limitation (Snow et al., 2015; Walworth et al., 2016, 2018). However, recent ‘omic studies of Fe or P physiology in *Trichodesmium* have typically surveyed gene-product dynamics using a single ‘omics tool, either transcriptomics (Polyviou et al., 2018) or proteomics (Snow et al., 2015; Walworth et al., 2016), and have only recently been applied in parallel (Walworth et al., 2018). It has been widely observed that mRNA and protein levels for specific genes are not always well correlated, leading to potentially disparate biological interpretations depending on the data products used (Palenik, 2015). Accounting for factors like post-transcriptional modification, disconnects between transcription and translation, and in the turnover times for transcripts and proteins is challenging and has incited caution about whether measuring gene expression or protein abundance is the most ecologically sound indicator of nutritional physiology in an organism (Palenik, 2015). As such, predicting physiology from only one molecular approach, either gene expression or protein abundance of specific biomarkers, may yield different interpretations when assaying *Trichodesmium* physiology, particularly in the field. Here, we examine the response of *Trichodesmium* to P deficiency and re-supply using parallel transcriptomic, proteomic, and enzyme activity assays. This integrative analysis aims to provide greater resolution of the dynamics of P stress biomarkers to increase their utility and application to field populations.

## Experimental Procedures

### Culture Conditions

Experiments were performed with *Trichodesmium erythraeum* IMS101, which was isolated from the coastal waters off North Carolina in 1991 (Prufert-Bebout et al., 1993). Cultures were maintained in 0.2 μm sterile-filtered YBC-II growth media, prepared as described in Chen et al. (1996), with modulation of the KH_2_PO_4_ concentration as described below. Cultures were grown at 24°C on a shaker table (80 rpms) in a 12:12 light:dark cycle (∼70 μmol quanta m^-2^ s^-1^). Under these conditions, *T. erythraeum* IMS101 was present in the form of free filaments. All cultures were uni-algal but not axenic.

### Experimental Design – P Stress and Re-supply

A phosphate replete (50 μM KH_2_PO_4_) culture was used to inoculate 1.5 L sterile YBC-II media prepared without phosphate (no added KH_2_PO_4_), herein referred to as -P, in each of nine 2 L baffled polycarbonate flasks. Three of the 9 flasks were amended with 50 μM KH_2_PO_4_ (herein referred to as +P). Growth was monitored daily by measuring *in vivo* phycoerythrin fluorescence using a Turner Designs Aquafluor handheld fluorometer (Supplementary Figure 1). P-deficient (-P) and replete (+P) cultures did not exhibit significant differences in growth rate (ANCOVA, *p* = 0.52; Supplementary Figure 1). When +P and -P replicates began to exhibit differences in fluorescence, daily measurements of alkaline phosphatase activity (APA; see methods below) commenced in order to ensure P stress (indicated by significant increase in APA in the -P treatment relative to replete). Measurements were made at the same time each day (∼ 3 h into the light phase). Triplicate +P replicates were harvested during late-log phase growth and 3 of the 6 (chosen randomly) -P replicates were harvested once APA was significantly increased relative to the +P treatment, herein referred to as *t* = 0 h. At this point, the remaining volume in the 6 -P treatments was pooled, mixed, and split out equally into 6 flasks (1.2 L per flask). Triplicate -P cultures were resupplied with P to replete conditions by adding 50 μM KH_2_PO_4_, herein referred to as RF. Both -P and RF cultures were subsampled at 4, 24, 48, and 72 h after the addition of P in the P-resupplied flasks. For this portion of the experiment, and including *t* = 0 h samples, quantification of cell numbers was performed using quantitative PCR (qPCR) (see method described below). All culture work and sampling was done in an Airclean 4000 laminar flow hood (Airclean Systems, Creedmoor, NC, United States) with a flame. The first experimental time point, *t* = 0 h, was taken at 10 a.m. local time, ∼3 h into the 12 h light period.

### Biochemical Analysis

APA was assayed to confirm P-deficiency prior to the start of the experiment and from triplicate biological samples from all time points (*t* = 0, 4, 24, 48, and 72 h). Samples (2 mL) were filtered onto 47 mm polycarbonate membranes (0.2 μm) and stored at -20°C until analysis. Activity was assayed as previously described (Dyhrman and Ruttenberg, 2006) using the fluorogenic phosphatase substrate 6,8-difluoro-4-methylumbelliferyl phosphate. APA values were compared across treatments using a student’s t-test.

### *Trichodesmium* Enumeration by qPCR

For all time point samples collected in this study, *Trichodesmium* cell abundance was monitored following a previously described qPCR protocol (Rouco et al., 2014). At the time of harvest, samples (5 mL) were filtered onto 25 mm 5 μm polycarbonate filters, flash frozen in liquid nitrogen, and stored at -80°C prior to extraction. DNA was extracted using a MoBio Power Plant Pro DNA isolation kit following manufacturer instructions (Qiagen, Hildern, Germany; Rouco et al., 2014) and the abundance of *Trichodesmium* was determined with qPCR using a *T. erythraeum* clade-specific primer set for the *rnpB* gene (Chappell and Webb, 2010). Amplification of standards, no template controls (RNase-free water), and time point samples (*t* = 0, 4, 24, 48, and 72 h) were run in triplicate on a Bio-Rad CFX96 Real-Time PCR detection system using Bio-Rad SYBR Green SuperMix (Bio-Rad Laboratories, Inc., Hercules, CA, United States). Standard curves were generated from DNA extracts performed on filters with known concentrations of *T. erythraeum* IMS101. Concentrations were previously determined by 10 replicates of cell counts using a Sedgwick Rafter slide (Rouco et al., 2014). Extraction efficiencies were assumed to be equivalent across samples and standards as per Rouco et al. (2014). Reactions were run in final volumes of 25 μL, with 12.5 μL SuperMix, 2 μL template, 9.5 μL sterile water, and 200 nmol (final concentration) forward and reverse primers. Reaction conditions were as follows: 2 min. at 50°C, 10 min. at 95°C; 40 cycles of 15 s at 95°C, and 1 min at 55°C with a fluorescence measurement. Resulting quantification cycle (Cq) values for the *rnpB* gene were averaged across triplicates and compared against the standard curve to calculate cell number.

### RNA Extraction, Sequencing, and Analysis

At the times of harvest, 25 mL subsamples of each culture flask were filtered onto polycarbonate filters (25 mm, 5 μm). The filters were immediately flash frozen and stored in liquid nitrogen. Prokaryotic RNA was extracted with the Qiagen RNeasy Mini Kit (Qiagen) with a minor modification to the lysis step. Briefly, ∼250 μl zirconia/silica beads (0.5 mm) were added to each sample tube before the addition of Buffer RLT, and the samples were vortexed for 5 min. The resulting lysate was processed as per the remainder of the manufacturer instructions, including on-column DNase digestion (RNase-free DNase Kit, Qiagen). Ribosomal RNA was removed using the Ribo-Zero Magnetic Kit for bacteria (Illumina, Inc., San Diego, CA, United States) as per manufacturer instructions. Purified prokaryotic mRNA was concentrated using the RNeasy MinElute Cleanup Kit (Qiagen) according to the directions provided by the manufacturer. The mRNA concentration and quality was assessed with a Bioanalyzer using the RNA 6000 Nano Kit (Agilent Technologies, Santa Clara, CA, United States). The Illumina TruSeq RNA sample preparation kit was used by the JP Sulzberger Genome Center at Columbia University (CUGC). Sequencing of 30 million, 100 base pair, paired end reads from each sample was performed on an Illumina HiSeq at the CUGC. Sequences were deposited in the NCBI SRA under accession number PRJNA429214.

Sequenced reads were trimmed using sickle^1^ and mapped to the *T. erythraeum* IMS101 gene models^2^ (Walworth et al., 2015) with RSEM and the default settings with the exception of using the paired end and bowtie2 options (Li and Dewey, 2011). Significant differential gene expression (*p* < 0.05 after false discovery rate correction (FDR) and fold change values between samples were determined using edgeR (McCarthy et al., 2012). Annotations of *T. erythraeum* IMS101 gene models were pulled from the UniProt database in order to coincide with proteomic analyses (described below). Translated proteins from the IMS101 gene models were screened for signal peptide motifs using SignalP 4.1 (Petersen et al., 2011), and for transmembrane domains using TMHMM v. 2.0.^3^

### Protein Extraction and Proteomic Analysis

At each time point, 95 mL subsamples of each culture flask were filtered onto polycarbonate filters (47 mm, 5 μm). The filters were immediately flash frozen and stored in liquid nitrogen. Protein extraction, digestion, and liquid chromatography mass spectrometry (LC-MS/MS) were carried out by the University of California at Davis Proteomics Core Facility (Davis, CA, United States). For protein extraction, filters were transferred to 2 mL tubes with 1.5 ml of extraction buffer (1% SDS, 0.1M Tris/HCl pH 7.5, 10 mM EDTA). All samples were incubated at room temperature (RT) for 15 min and heated at 95°C for 10 min with gentle shaking (350 rpm). Samples were then shaken (350 rpm) at RT for 1 h. The samples were centrifuged at 14,100 *g* (14,500 rpm) for 20 min at RT, and the supernatants were pipetted off and concentrated by centrifugal filtration to ∼ 300 μl with an Amicon filter unit (1 ml, 3 K molecular weight cutoff) (MilliporeSigma, Burlington, MA, United States). Total proteins in each sample were quantitated using the bicinchoninic acid (BCA) assay (Smith et al., 1985). Samples were precipitated with the ProteoExtract Protein Precipitation Kit (CalBiochem), according to the manufacturer’s instructions. Pellets were air-dried for 10 min at RT. Proteins were then reduced, alkylated and urea Lys-C/trypsin digested. Briefly, pellets (150 μg) were reconstituted in ∼100 μl 6 M urea in 50 mM ammonium bicarbonate (ambic). Proteins were reduced by adding dithiothreitol (DTT) in ambic to a final concentration of 5 mM and incubating for 30 min at 37°C. Proteins were alkylated by adding iodoacetamide in ambic to a final concentration of 15 mM and incubating for 30 min at RT in the dark. The alkylation was quenched with the addition of DTT (40 mM, final concentration) and incubation for 10 min at RT. Proteins were in-solution digested by adding MS-grade Lys-C/trypsin (Promega, Madison, WI, United States) in a 1:25 (enzyme:protein) ratio and incubating for 4 h at 37°C. Ammonium bicarbonate (50 mM) was added to dilute the urea and activate the trypsin. Proteins were digested overnight at 37°C and desalted and purified using MacroSpin columns (The Nest Group, Southborough, MA, United States), according to the manufacturer’s protocol.

Digested peptides were analyzed by LC-MS/MS on a Q Exactive Plus Orbitrap Mass spectrometer (Thermo Scientific) in conjunction with a Proxeon Easy-nLC II HPLC (Thermo Scientific) and Proxeon nanospray source. The digested peptides were loaded on a 100 μm × 25 mm Magic C18 100 Å 5U reverse phase trap where they were desalted online before being separated using a 75 μm × 150 mm Magic C18 200 Å 3U reverse phase column. Peptides were eluted using a 140 min gradient with a flow rate of 300 nl min^-1^. An MS survey scan was obtained for the m/z range 300–1600 and MS/MS spectra were acquired using a top 15 method, where the top 15 ions in the MS spectra were subjected to high energy collisional dissociation (HCD). An isolation mass window of 1.6 m/z was used for the precursor ion selection and normalized collision energy of 27% was used for fragmentation. A 15 s duration was used for the dynamic exclusion.

Tandem mass spectra were extracted and charge state deconvoluted by Proteome Discoverer [(Thermo Scientific). All MS/MS samples were analyzed using X! Tandem (The GPM, thegpm.org; version Alanine (2017. 2. 1.4))]. X! Tandem was set up to search the UniProt *Trichodesmium erythraeum* database (March 2017, 4355 entries), the cRAP database of common laboratory contaminants^4^ (114 entries), plus an equal number of reverse protein sequences assuming the digestion enzyme trypsin. X! Tandem was searched with a fragment ion mass tolerance of 20 PPM and a parent ion tolerance of 20 PPM. Iodoacetamide derivative of cysteine was specified in X! Tandem as a fixed modification. Deamidation of asparagine and glutamine, oxidation of methionine and tryptophan, sulphone of methionine, tryptophan oxidation to formylkynurenin of tryptophan and acetylation of the n-terminus were specified in X! Tandem as variable modifications.

Scaffold (version Scaffold_ 4.8.4; Proteome Software Inc., Portland, OR, United States) was used to validate MS/MS-based peptide and protein identifications. Peptide identifications were accepted if they exceeded specific database search engine thresholds. X! Tandem identifications required at least -Log (Expect Scores) scores of greater than 1.2 with a mass accuracy of 5 ppm. Protein identifications were made using the Protein Prophet algorithm in Scaffold 4.0 (Proteome Software, Portland, OR, United States) using 99% protein and 95% peptide confidence levels, allowing 1 minimum peptide per protein, resulting in a 5.2% false discovery rate for proteins and a 0.12% false discovery rate for peptides resulting in 1933 identified proteins. Proteins that contained similar peptides and could not be differentiated based on MS/MS analysis alone were grouped to satisfy the principles of parsimony.

Changes in relative abundance of proteins between samples were determined using label-free spectral count enumeration within Scaffold. Significance was determined using Fisher’s Exact test (*p* < 0.05). Spectral counts compare a specific protein’s relative abundance between treatments, rather than against other proteins, because the sensitivity of spectral counts can vary between proteins depending on the number of tryptic peptides within the sequence and their chemical characteristics. This does not affect comparisons of a protein with itself between treatments. Spectral counts were normalized within each experimental treatment to the total number of spectra collected to correct for small variations in the number of spectra between samples sets. Spectral libraries were deposited in the MassIVE repository (massive.ucsd.edu) under ID MSV000082959.

## Results and Discussion

Metatranscriptome and metaproteome profiling are important tools for screening phytoplankton *in situ* and interpreting phenotype and putative activities in a species-specific manner. Here we examined the response of *Trichodesmium* to P deficiency and re-supply using integrative analysis of the transcriptome and proteome to provide greater resolution of the dynamics of P stress and to provide baseline validation of these ‘omic approaches for their application to field populations.

### Responses to P Deficiency

**FIGURE 1:**
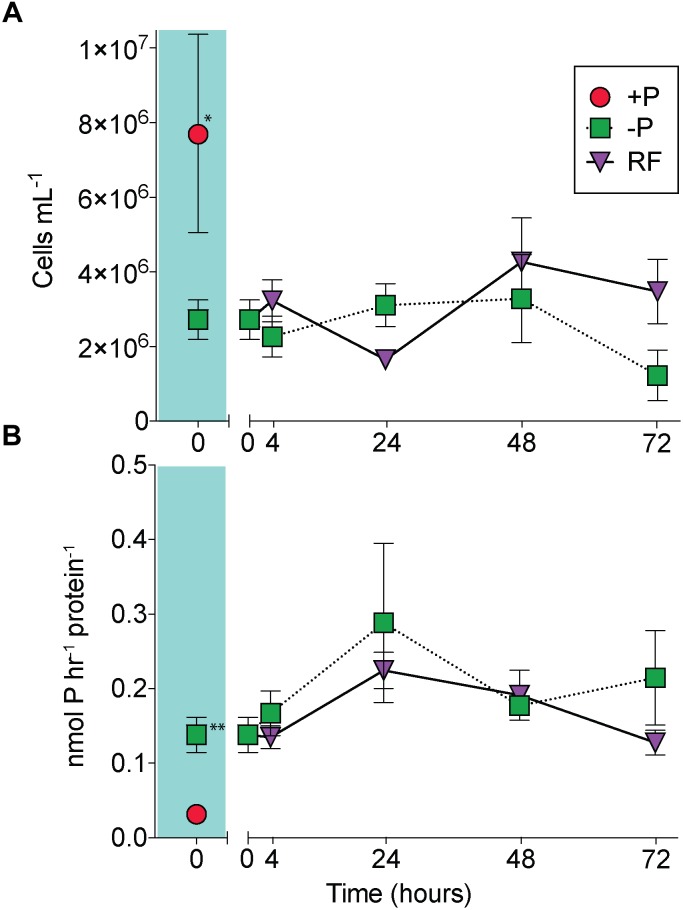
A comparison of *Trichodesmium* responses to phosphorus replete (+P), phosphorus deficient (–P) and phosphorus re-supply (RF) treatments. *Trichodesmium* cell abundance **(A)** and alkaline phosphatase activity (APA) **(B)** in +P (red circle), –P (green squares), and RF (purple triangles) across the 72 h study. The +P (*t* = 0 h) and –P (t = 0 h) comparison is shaded in teal. Asterisks indicate significantly different (unpaired *t*-test, ^∗^*p* = 0.02, ^∗∗^*p* = 0.006). Error bars represent standard error of the mean (*n* = 3).

At the onset of the experiment, *Trichodesmium* cell numbers were significantly reduced in the -P cultures relative to the +P cultures (Figure 1A; unpaired *t*-test, *p* = 0.02), and APA was significantly greater in the -P cultures compared to the +P (Figure 1B; unpaired *t*-test, *p* = 0.006). APs are capable of hydrolyzing ester-bound phosphate from organic P compounds, and their induction is a noted marker of P stress in phytoplankton, including *Trichodesmium* (Stihl et al., 2001; Dyhrman et al., 2002; Dyhrman, 2016). The significant differences in cell concentration and induction of APA relative to the +P control suggests that the -P cultures were experiencing P stress. The sustained presence of APA and consistent cell numbers in the -P samples (Figure 1) indicate prolonged P stress in these treatments over the 72 h study. In the RF cultures that had phosphate added, cell abundance increased relative to the -P cultures, but this increase was not significant at 72 h (Figure 1B), as there was an apparent lag in growth response in the RF treatment. In the RF treatment APA was reduced relative to the -P treatment at 72 h, but this difference was not significant (Figure 1B). This lagged response in growth and APA with P addition is consistent with previous work (Orchard et al., 2009). *Trichodesmium* APs do not appear to be actively degraded or intracellularly recycled as there was also a sustained (48 h) period without significant reduction in APA following P re-supply in that study (Orchard et al., 2009).

**FIGURE 2:**
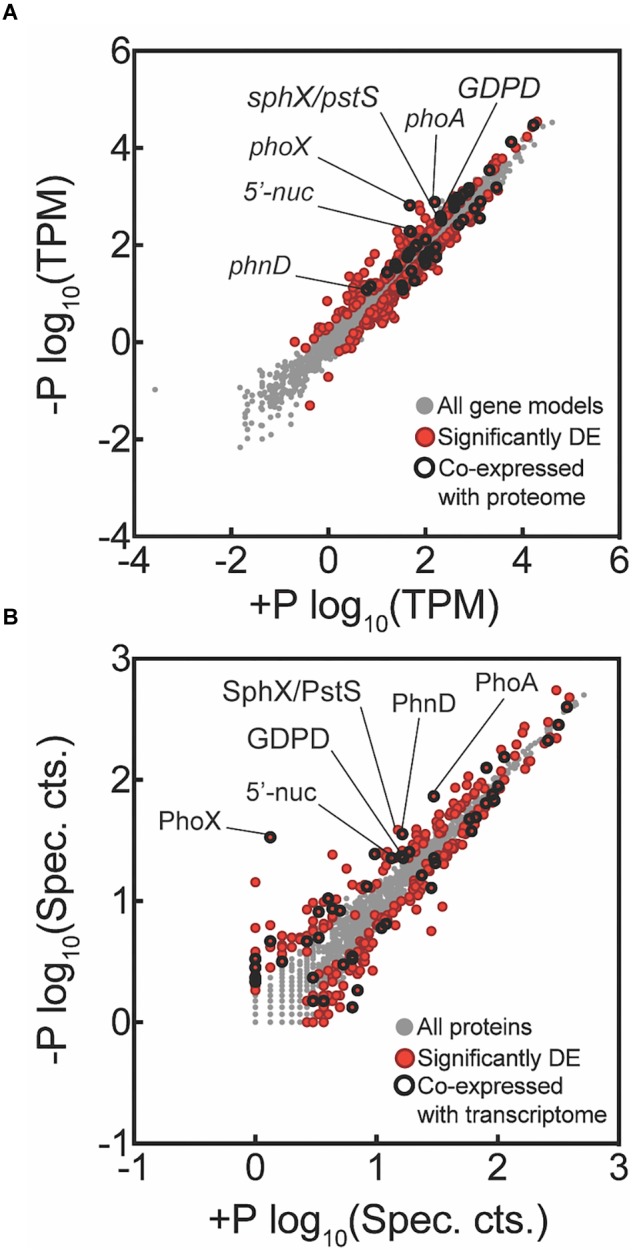
Global transcriptome and proteome signals from *Trichodesmium* comparing phosphorus replete (+P, *t* = 0 h) and phosphorus-deficient (–P, *t* = 0 h) samples. Each data point represents a unique gene **(A)** or protein **(B)**, with those that are significantly differentially expressed (DE) indicated in red. Data points that were significantly different and co-expressed in both the transcriptome and proteome are highlighted with a black circle. For the transcriptome data, tags that mapped to a gene model were summed and then the total count was normalized to library size in tags per million (TPM) and significance was determined with edgeR (*p* < 0.05 after false discovery rate (FDR) correction). For the proteome data, spectral counts were normalized within each experimental treatment to the total number of spectra collected to correct for small variations in the number of spectra between samples sets. Differential abundance of proteins was determined using the Fisher’s Exact test, *p* < 0.05. Key genes are called out with text. All key genes called out exhibited significantly increased expression or abundance in both transcriptome and proteome, with the exception of SphX/PstS.

Transcriptome reads were mapped to the 4451 protein-coding genes (625 pseudo-genes removed) of *Trichodesmium erythraeum* IMS101 available on IMG. Of this total, 4436 genes had one or more reads mapped from across all samples, indicating detection of over 99% of the protein-coding gene models. There were 499 genes significantly differentially expressed between the -P and +P treatments at *t* = 0 h (*p* < 0.05 after FDR correction; Figure 2A). Of these differentially expressed genes, 233 had increased expression in -P while the remaining 266 had decreased expression in -P. The quantitative global shotgun proteomic analyses conducted in parallel with the transcriptome profiling recovered 1933 proteins from 1,340,670 mass spectra. This represents 43% of the 4451 protein-coding genes available on IMG and is a level of coverage similar to other proteomic studies with *Trichodesmium* (e.g., Walworth et al., 2016). After spectral count enumeration and count comparison with Scaffold, 286 proteins were found to have significantly different abundance between the -P and +P treatments (Fisher’s Exact test, *p* < 0.05; Figure 2B). Of these differentially abundant proteins, 133 were increased in -P while the remaining 153 were decreased. A complete list of gene expression counts and relative protein abundances (spectral counts) are included in the Supplementary Data Set.

**FIGURE 3:**
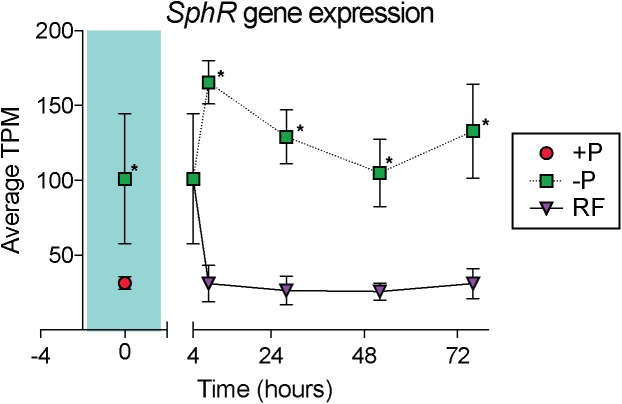
Gene expression dynamics of the *sphR* gene, a putative transcriptional response regulator that may control expression of genes in the pho regulon. The teal shaded area indicates comparison between phosphorus replete (+P) and phosphorus deficient (–P) treatments at *t* = 0 h, subsequent points compare –P and phosphorus re-supply (RF) treatments. Asterisks indicate significantly different expression (*p* < 0.05 after false discovery rate (FDR) correction) between treatments.

In cyanobacteria, conserved P stress responses typically include the upregulation of proteins involved in the high affinity uptake of phosphate, and the uptake and hydrolysis of DOP compounds (e.g., Martiny et al., 2006; Orchard et al., 2009; Teikari et al., 2015). The genes making up this P stress response are often referred to as the pho regulon, after the term described for *Escherichia coli* (Torriani-Gorini, 1987; Wanner and Low, 1996). Transcription of the *Trichodesmium* pho regulon is thought to be controlled by the transcriptional regulator SphR, an ortholog of *E. coli* PhoB (Su et al., 2007). SphR binds to specific regions of DNA upstream of pho regulon genes, called pho boxes. Pho boxes have been identified upstream of a number *Trichodesmium* genes including the gene encoding the high affinity phosphate binding protein, *sphX* (Tery_3534), the gene encoding the phosphonate binding protein, *phnD* (Tery_4993), and the alkaline phosphatase genes *phoX* (Tery_3845) and *phoA* (Tery_3467) (Su et al., 2007; Orchard et al., 2009) (Table 1). Here, *sphR* (Tery_2902) gene expression was significantly higher in the -P treatment relative to the +P control (*t* = 0 h), and was reduced to +P control levels within 4 h of P re-supply (Figure 3). This gene expression pattern is consistent with its putative regulatory role in the P stress response. SphR was not detected in the proteome (Table 1), however regulators may have low abundance relative to other proteins owing to rapid production and degradation (Vogel and Marcotte, 2012). Coincident with its putative role in the pho regulon, *sphR* expression in the –P treatment (*t* = 0 h) was co-expressed with pho box-adjacent genes *sphX, phnD, phoX* and *phoA*, all of which were significantly increased in the –P treatment relative to the control, with the exception of *sphX*, which was not significantly different until *t* = 24 h (Table 1 and Figure 2A). Furthermore, the proteins for these genes were all significantly more abundant in the -P treatment relative to the +P treatment (*t* = 0 h) (Figure 2B). We note that at the amino acid level portions of SphX (Tery_3534) and PstS (Tery_3537) are similar, and we were unable to distinguish between them with the peptides identified by our proteomics method. Given that *pstS* was not P-regulated in previous work (Orchard et al., 2009), or here (Supplementary Data Set), we refer to the protein product as SphX for clarity. Taken together, the presence of putative pho boxes, and their consistent regulation with *sphR*, suggests that these genes and their concomitant activities make up the pho regulon in *Trichodesmium*.

**Table 1:** Transcript and protein signals for putative pho regulon genes at *t* = 0 h (-P_t = 0 h_ vs. +P_t = 0 h_) and t = 24 h (-P_t = 24 h_ vs. +P_t = 0 h_).

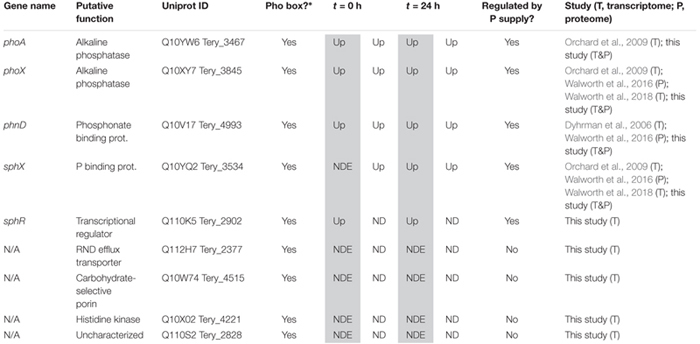

*The shaded column denotes the transcriptome, while unshaded column denotes the proteome. Significance was determined with edgeR (transcripts, *p* < 0.05 after false discovery rate correction) or Fisher’s Exact test (proteins, *p* < 0.05). Up, greater significance in the –P relative to the +P (*t* = 0 h) or the -P (*t* = 24 h) relative to +P. NDE, not differentially expressed. ND, not detected.*

Su et al. (2007) also identified pho boxes associated with genes in *Trichodesmium* putatively related to transport or signaling [an RND family efflux transporter (Tery_2377), a carbohydrate-selective porin (Tery_4515), a PAS/PAC sensor signal transduction histidine kinase (Tery_4221), and an uncharacterized protein with putative kinase domains (Tery_2828)], leading to the speculation that these genes could be P-regulated and play a role in the P stress response. In this dataset, however, none of these genes had significantly increased expression in the -P treatment (*t* = 0 h) relative to the +P treatment, and they were not detected in the proteome (Table 1). These gene targets do not appear to be a part of the canonical pho regulon for *Trichodesmium*.

Literature comparisons across different *Trichodesmium* culture studies can be challenging because differences in culture medium and experimental design make it difficult to constrain the degree of P deficiency. Despite these challenges, there is a striking degree of concordance between the putative pho regulon gene expression and protein abundance with the signals from other studies (Table 1). Both targeted assays and global transcriptome sequencing have identified increased gene expression of *sphX, phoX*, and *phoA* (Orchard et al., 2009; Walworth et al., 2018), and *phnD* (Dyhrman et al., 2006), and proteomics identified increased abundance of the SphX protein (Walworth et al., 2016) with increasing P stress in P deficient culture studies with *T. erythraeum* IMS101, consistent with those observed here (Table 1). In *Trichodesmium*, the genes downstream of *phnD* are also involved in phosphonate metabolism and transport and have been observed to be P-regulated, with P deficiency increasing the gene expression of *phnJ* in a targeted gene expression study (Dyhrman et al., 2006), and increasing the relative protein abundance of PhnG, PhnK, PhnL, and PhnM in a recent proteome study (Walworth et al., 2016). Here, we also observed significantly increased protein abundance of PhnM in the -P treatment (*t* = 0 h) relative to the +P control (Supplementary Data Set), however the other proteins were not detected. The associated expression signals for these genes were low (typically < 10 TPM on average across the triplicate samples), making them difficult to resolve, although *phnJ* was significantly increased in the -P treatment (*t* = 24 h) relative to the +P treatment (Supplementary Data Set). These patterns all suggest that *Trichodesmium* coordinates expression of the genes that encode the machinery for transporting and metabolizing phosphonate together, and that the expression of this cassette of genes is under the control of the pho regulon. The consistent response observed across studies suggests that *phnD*, and to a lesser extent *phnJ* or *phnM*, are possible biomarkers of the process of phosphonate transport and hydrolysis in field populations. The expression of genes related to phosphite uptake (*ptxB*: Tery_0366) and metabolism (*ptxD*: Tery_0368) have also been shown to be both P-regulated and substrate responsive in *T. erythraeum* IMS101 using a targeted qRT-PCR approach (Polyviou et al., 2015). Notably, in the *T. erythraeum* IMS101 genome these genes do not occur adjacent to a pho box and thus are not expected to be under canonical pho regulon control. In this study, both transcripts and proteins for these genes were detected but they were not significantly increased in the -P treatment (*t* = 0 h) relative to the +P treatment (Supplementary Data Set). The differences observed here relative to other studies tracking the metabolism of reduced P may be related to differences in the relative degree of P stress between experiments, or differences in the sensitivity of targeted versus global screening methods, among other possibilities.

In addition to the canonical P stress response of the pho regulon, there were a number of genes with potential roles in P physiology that were significantly increased in the -P (*t* = 0 h) transcriptome and proteome relative to the +P treatment. An enzyme with putative nucleotidase/exopolyphosphatase activity (Tery_1774) was significantly increased in the transcriptome and proteome in -P treatments (*t* = 0 h) relative to the replete control (Figure 2). The protein sequence of this enzyme does not have a clear signal peptide or transmembrane domain, suggesting that P might be scavenged from intracellular P containing metabolites, such as nucleotides, or polyphosphate, when *Trichodesmium* is P-stressed. *Trichodesmium* and other phytoplankton are known to accumulate polyphosphate in low P regions (Orchard et al., 2010; Martin et al., 2014), and this enzyme may play a role in scavenging P from this potential P store. Nucleotidases can exhibit increased gene expression during low P conditions in model bacteria (Baek and Lee, 2006). Furthermore, in eukaryotic marine phytoplankton, these genes are also often P-regulated (Wurch et al., 2011; Dyhrman et al., 2012; Frischkorn et al., 2014) and have been shown to modulate the ratio of AMP:adenosine, scavenging P from AMP when cells are P-stressed (Kujawinski et al., 2017). In *Trichodesmium* this enzyme may also be driving a similar response. However, in bacterioplankton, 5′-nucleotidase activity is not typically regulated by P (Ammerman and Azam, 1985), so more work is required to understand the role of this enzyme in the *Trichodesmium* P stress response.

*Trichodesmium* has been shown to substitute membrane phospholipids like phosphatidylglycerol (PG) for the sulfur-containing lipid sulphoquinovosyldiacylglycerol (SQDG) when P-stressed, a switch that can substantially reduce cellular P quotas (Van Mooy et al., 2009). Data from other organisms suggests this involves the biosynthesis of SQDG, and the breakdown of PG (Martin et al., 2011; Iwai et al., 2014). Consistent with this known adaptation to P deficiency, the abundance of the SqdB enzyme (Tery_0398) that synthesizes SQDG was significantly increased in the -P proteome relative to the +P treatment (Figure 2A). Although a significant increase in *sqdB* gene expression was not detected in the -P transcriptome at *t* = 0 h, it was significantly increased by the *t* = 24 h timepoint (Supplementary Data Set). In addition, a glycerophosphoryl diester phosphodiesterase enzyme (GDPD: Tery_2777), an enzyme that hydrolyzes phosphodiester bonds from phospholipid molecules like PG, was significantly increased in the -P (*t* = 0 h) transcriptome and proteome relative to the +P treatment (Figure 2A). This gene may be involved in breaking down phospholipids. In brief, these patterns and their underlying functional roles suggest that these enzymes may underpin the lipid substitution that happens in P-stressed *Trichodesmium*.

### Transcriptomic and Proteomic Choreography in Response to P Deficiency

**FIGURE 4:**
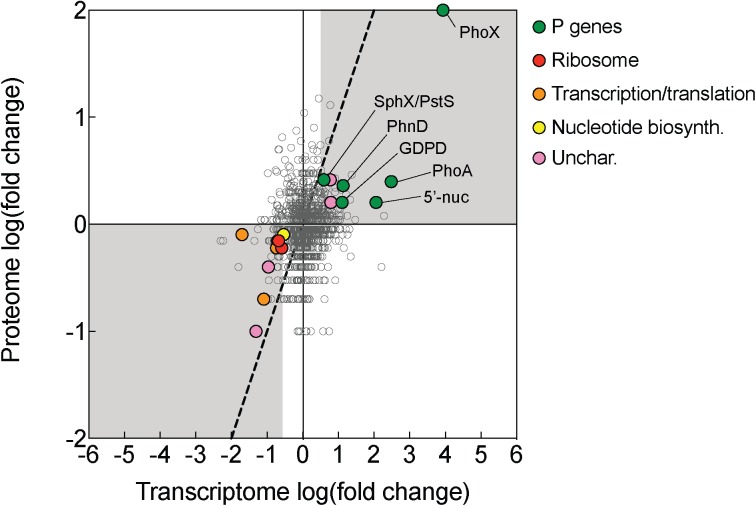
Transcriptomic and proteomic choreography in specific protein categories in response to P deficiency. Fold change presented as the log_2_ of the ratio of phosphorus replete (+P; *t* = 0 h): phosphorus deficient (–P; *t* = 0 h) conditions. A 1:1 line (dashed) denotes equal fold change between the two conditions in the proteome and transcriptome. Genes/proteins with significant differences in expression or abundance were grouped according to functional category and color-coded: P genes (exhibited evidence of P-regulation in this study), ribosomes, transcription/translation, nucleotide biosynthesis (nucleotide biosyn.), and uncharacterized (unchar.) proteins.

Of the total differentially expressed genes and proteins, 25 were jointly increased in the -P treatment, and 23 were jointly decreased at *t* = 0 h (Supplementary Table 1). Genes of the pho regulon, and other genes highlighted herein, exhibited strikingly consistent choreography between the transcriptome and the proteome (Figure 2, 4), suggesting that induction of these signals as a population becomes P-stressed is tightly coordinated. Congruent patterns of gene expression and protein abundance in response to P deficiency have also been demonstrated in other phytoplankton, including other N_2_ fixing cyanobacteria, diatoms, and in the pelagophyte *Aureococcus anophagefferens*, as well as higher plants, grown under P-deficient conditions (Wurch et al., 2011; Dyhrman et al., 2012; Lan et al., 2012; Cox and Saito, 2013; Teikari et al., 2015). Taken together, these signals reinforce what is known about the adaptive strategy to P deficiency in *Trichodesmium*, a strategy involving increased phosphate uptake, a reduction in P quota, and the use of DOP.

There was also notable choreography between significantly downregulated genes and proteins reflective of broad metabolic restructuring in response to low P. Functions related to ribosome synthesis, transcription and translation, and nucleotide biosynthesis represented functional categories that were most strongly downregulated in -P relative to replete in both the transcriptome and the proteome (Figure 4). This response could be connected to differences in growth patterns in response to -P conditions, which is reflected in the lower cell yield in the -P culture relative to +P seen at *t* = 0 h (Figure 1A). In *Trichodesmium*, genes related to translation/ribosomes exhibited significantly decreased expression in P-stressed treatments relative to P replete at both modern and elevated CO_2_ (Walworth et al., 2018), supporting the idea that this physiological shift is consistently observed across independent studies with different culture conditions. Such patterns are also similar to those observed in coupled transcriptome/proteome studies in *Synechococcus*, *T. pseudonana* and *A. anophagefferens* (Wurch et al., 2011; Dyhrman et al., 2012; Cox and Saito, 2013). Ribosomes themselves are rich in P (Elser et al., 2010), and RNA synthesis is also a significant biochemical drain of this resource (Van Mooy and Devol, 2008). As such, the downregulation of ribosomal proteins and the concurrent repression of transcription and translation observed in *Trichodesmium* (Figure 4) may serve as a P conservation strategy under P deficient conditions (Grosse et al., 2017 and references therein). Because of the scope of our experimental design, we could not disentangle if these patterns are specific to P deficiency or serve as an overarching response to general cellular stress similar to the stringent response that is widespread in bacteria (Potrykus and Cashel, 2008). In other species, genes encoding functions related to RNA processing and ribosomes were downregulated in response to growth in low nitrogen conditions (Harke and Gobler, 2013), suggesting that in cyanobacteria the expression of genes encoding these functions could be related to stress in general. However, in *Trichodesmium*, the gene expression of translation/ribosome functions had a different pattern under Fe deficiency than under P deficiency (Walworth et al., 2018). This response indicates that some stress responses may vary by the type of stressor, and more work is needed to identify how metabolism is restructured in *Trichodesmium* and to evaluate shared and disparate responses across a range of conditions.

While most of the coordinated responses observed in the transcriptome and proteome were attributable to increases in processes related to organic P hydrolysis, P transport, and decreases in P-rich growth-related functions, several uncharacterized proteins also displayed significant choreography in response to low P (Figure 2, 4). These uncharacterized proteins were observed to be both induced and repressed in -P in both the transcriptome and proteome (Figure 3). This coordination hints at aspects of P physiology that are yet to be characterized in *Trichodesmium*, but may be important for generating a more complete understanding of how this diazotroph thrives in nutrient-poor oligotrophic ocean ecosystems.

Of the 499 total significantly differentially expressed genes in the -P treatment (*t* = 0 h), only 240 of the corresponding protein products were detected in the proteome (Supplementary Data Set). As such, ∼48% of the significant responses detected through transcriptional analysis were not detected through proteomic screening of samples taken in parallel. Furthermore, only ∼10% of those proteins (*n* = 48) detected in parallel with the transcriptome also exhibited significant differential abundance, or co-expression, between treatments (Supplementary Table 1). This largely reflects the fact that fewer proteins are detected overall in the proteome and highlights potential discrepancies in physiological interpretations that could arise based on experimental design. There are many factors which can cause this type of disconnect between the transcriptome and proteome. These factors may be methodological, deriving from differences in the relative sensitivities of RNA-seq versus proteomic approaches or from not using the same sample for each approach (Nie et al., 2007). Discord between gene expression and protein abundance may also stem from the level of stress, as high stress has been shown to yield higher correlation (Halbeisen and Gerber, 2009; Waldbauer et al., 2012). Furthermore, mRNA and proteins that are highly abundant have been shown to be more strongly correlated (Nie et al., 2007; Fan et al., 2017). Finally, the stability of mRNA relative to proteins within cells could play a role in observed discordance. The half-life of proteins is typically days, while mRNA is hours (Maier et al., 2009; Vogel and Marcotte, 2012 and references therein), and this could also contribute to a lack of co-expression.

### Decoupled Physiological Response to P Re-supply

**FIGURE 5:**
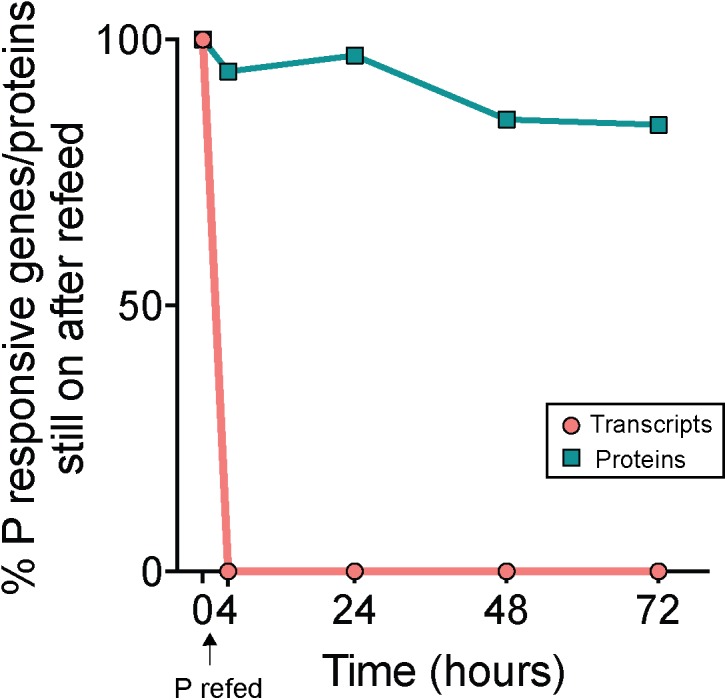
Decoupled physiological response to phosphorus re-supply in the transcriptome and proteome. Percentages indicate the proportion of genes or proteins that originally had significantly increased expression or abundance in phosphorus deficient (–P; *t* = 0 h) treatments relative to phosphorus replete (+P; *t* = 0 h) treatments that still exhibited significantly increased expression or abundance following re-supply of P (RF) at subsequent time points. Note the rapid decline in gene expression (<4 h) relative to the protein abundance, indicating a rapid turnover of transcripts but not proteins with P addition.

P was added to -P cultures to assess transcript and protein turnover in response to P re-supply (RF treatment). Notably, 100% of the total genes that initially had significantly upregulated expression in -P relative to +P at *t* = 0 h (*n* = 233, Supplementary Data Set) were no longer significantly upregulated 4 h following P re-supply (Figure 5). This indicates a complete transcriptional turnover in response to P supply over rapid time scales. This trend was not mirrored in the proteome. Compared to the complete turnover of P-responsive transcripts, 94% of the proteins that significantly increased in abundance in -P at *t* = 0 h (*n* = 133, Supplementary Data Set) were still significantly more abundant 4 h after P re-supply (Figure 5). Even after 72 h, 84% of the proteins from the initial set detected at *t* = 0 h were still significantly more abundant in the RF treatment relative to +P (Figure 5). These data suggest that the proteins of the P stress response were not actively degraded with P re-supply. With a much longer turnover time than transcripts, the proteome appears to reflect the nutrient history of the cell better than the instantaneous physiology. A lack of concordance between the transcriptome and proteome is not uncommon, and has been observed in response to perturbations in model bacteria like *E. coli* (e.g., Lee et al., 2003), and in marine bacterioplankton like *Prochlorococcus* and *Pelagibacter ubique* (Smith et al., 2010; Waldbauer et al., 2012). In *Prochlorococcus* cultures grown under non-stress conditions, daily oscillations in the abundance of the majority of detectable proteins were minimal when compared to the magnitude of shifts in the expression of the genes encoding them (Waldbauer et al., 2012). In the environment, *Trichodesmium* blooms form rapidly, accumulating up to 10,000 trichomes L^-1^ over expansive areas of the ocean, and can decline just as suddenly with concentrations decreasing by half less than 1 day following bloom peak (Capone et al., 1998; Rodier and Le Borgne, 2008; Spungin et al., 2016). Over these abrupt bloom-and-bust time scales, the turnover time of protein-level biomarkers may not be sensitive enough to accurately gauge physiological changes underpinning growth or decline.

**FIGURE 6:**
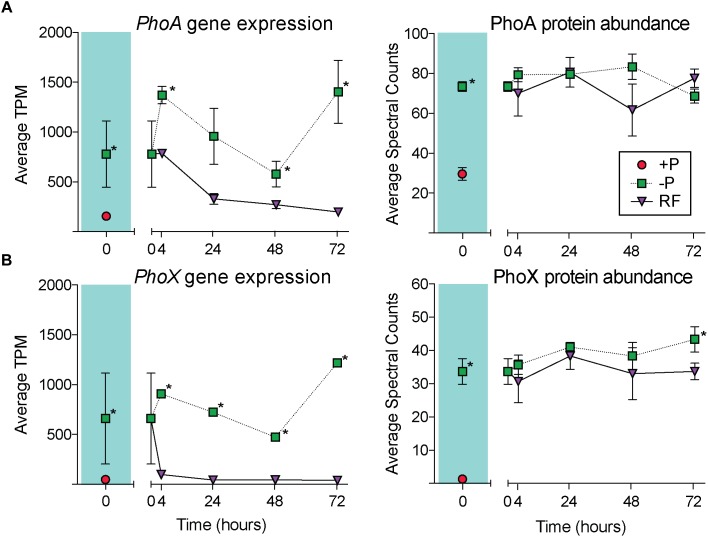
Gene expression and protein abundance patterns in the alkaline phosphatases, PhoA and PhoX. The dynamics of PhoA across the course of the experiment in the transcriptome **(A)** and proteome **(B)**, with the phosphorus replete (+P; *t* = 0 h) and phosphorus deficient (–P; *t* = 0 h) comparison shaded in teal. The dynamics of PhoX across the course of the experiment in the transcriptome **(C)** and proteome **(D)**, with the +P (*t* = 0 h) and –P (*t* = 0 h) comparison shaded in teal. Asterisks indicate significantly different gene expression between treatments after edgeR analysis (*p* < 0.05 after false discovery rate (FDR) correction) or significantly differential protein abundance (Fisher’s Exact test, *p* < 0.05).

Gene expression and protein abundance choreography in PhoA and PhoX show that these canonical P stress response signals in *Trichodesmium* followed the discordant pattern of the transcriptome and proteome datasets as a whole (Figure 6). As previously discussed, in both the transcriptome and proteome PhoA and PhoX were statistically significantly upregulated in the -P cultures relative to the +P controls (*t* = 0 h), a result that was supported by increased APA (Figure 1, 2). In the RF cultures, P re-supply resulted in rapid depletion of the *phoA* and *phoX* transcripts, decreasing significantly over the 72 h sampling period to levels that were similar to the level of expression in the original +P cultures (Figure 6). In contrast, the abundance of PhoA and PhoX proteins remained stable from the point of P re-supply, with the only significant difference between -P and RF occurring in PhoX at 72 h post-re-supply (Figure 6). Again, these proteins track with APA upon P re-supply and do not appear to be actively degraded (Figure 6). The persistence of these proteins for several days after P re-supply complicates the use of these, and the other pho regulon proteins, as biomarkers of a P stress phenotype, particularly in an environment with variable P, or boom-bust cycles like those highlighted above. However, the protein better tracks with its associated activity (APA) than gene expression after P re-supply. Thus, protein abundances are arguably better indicators of activity than gene expression in this context. In summary, assessing gene expression provides an important measure of phenotype, with insight into nutrient conditions at the exact time of sampling, while protein levels might be better suited to assessing the physiological history of the population and tracking activities. Similar studies performed with a range of nutrients, other stressors (e.g., light intensity and *p*CO_2_), and multiple stressors applied in combination, would build on how to most accurately interpret *Trichodesmium* metatranscriptome and metaproteome data, and this study underscores the value of applying both approaches to evaluate physiological ecology *in situ*.

## Conclusion

Metatranscriptome and metaproteome analyses are increasingly important tools for tracking microbial physiology and biogeochemistry in the field, and identifying how the transcriptome and proteome are modulated in control cultures can help interpret these data. This study identified a putative pho regulon likely under *sphR* control. As cells become P deficient, the strong choreography between gene expression and protein abundance in the pho regulon highlights the diverse strategies *Trichodesmium* uses to maintain growth and N_2_ fixation in low P environments, including accessing organic and inorganic P sources from the environment and the turnover of intracellular P stores. These results also highlight the potential utility of transcriptomes and proteomes in inferring drivers of these processes. However, the differences in turnover time between transcripts and proteins observed here with P re-supply must be taken into consideration, particularly in environments with variable P. For *Trichodesmium* field studies, measuring biomarkers at the transcript level would provide an accurate picture of P stress, as known P-responsive signals were largely co-expressed between the transcriptome and proteome during P stress, but became decoupled after P re-supply. Conversely, with the noted choreography here between proteins and activities, even with P re-supply, measuring proteins would provide a robust indicator of the presence of a given activity. This validation of ‘omic approaches provides important context for tracking the physiology of *Trichodesmium in situ*, particularly for interpreting how resources like P drive growth and N_2_ fixation in the field. However, more experimentation in the laboratory and on field samples will be necessary to fully integrate metatranscriptome or metaproteome data into modeling efforts focused on *Trichodesmium* biogeography and biogeochemistry. As oligotrophic ocean conditions expand in response to climate change (Polovina et al., 2008; Irwin and Oliver, 2009), so too might the environments in which *Trichodesmium* experiences potentially P-limiting conditions, making such approaches even more critical.

## Data Availability

The transcriptome sequences generated for this study can be found in NCBI SRA, PRJNA429214. Protein datasets are deposited in the MassIVE repository (massive.ucsd.edu) under ID MSV000082959.

## Author Contributions

SD, SH, and KF designed the experiments. SH and KF carried out the experiment and analyzed the data. SD, SH, and KF wrote the manuscript.

## Conflict of Interest Statement

The authors declare that the research was conducted in the absence of any commercial or financial relationships that could be construed as a potential conflict of interest.

## Footnotes

1 https://github.com/najoshi/sickle

2 https://genome.jgi.doe.gov/portal/trier/trier.home.html

3 http://www.cbs.dtu.dk/services/TMHMM/

4 www.thegpm.org/crap
