# Supplementary material for: Transcriptional and Proteomic Choreography Under Phosphorus Deficiency and Re-supply in the N2 Fixing Cyanobacterium Trichodesmium erythraeum
Source: Front Microbiol. 2019 Mar 5;10:330. doi: 10.3389/fmicb.2019.00330 (PMC6411698; doi:10.3389/fmicb.2019.00330)
Supplement: Supplementary file 1 [file Table_1.docx]

**Supplementary Table 1.** Annotations of genes/proteins that were significantly differentially expressed in both the transcriptome and proteome.

| **Co-expressed Annotations (down)** | **Tery ID*** | **Uniprot ID** | **Co-expressed Annotations (up)** | **Tery ID*** | **Uniprot ID** |
| --- | --- | --- | --- | --- | --- |
| 5-methyltetrahydropteroyltriglutamate--homocysteine methyltransferase | metE | Q117R7 | 4-hydroxybenzoyl-CoA thioesterase | Tery_4502 | Q10W86 |
| 50S ribosomal protein L1 | rplA | Q119S7 | 5'-nucleotidase / 3'-nucleotidase / exopolyphosphatase | Tery_1774 | Q114P0 |
| 50S ribosomal protein L11 | rplK | Q119S6 | Acetylornithine aminotransferase | argD | Q111I1 |
| Adenine phosphoribosyltransferase | apt | Q119D0 | Adenylyl-sulfate kinase | cysC | Q113Z6 |
| Adenylosuccinate synthetase | purA | Q10ZD0 | Alkyl hydroperoxide reductase/ Thiol specific antioxidant/ Mal allergen | Tery_0235 | Q119V1 |
| ATP-dependent Clp protease adapter protein ClpS | clpS | Q10Y18 | Glycerophosphoryl diester phosphodiesterase | Tery_2777 | Q110W5 |
| Bifunctional protein PyrR | pyrR | Q114G0 | HAD superfamily (Subfamily IA) | Tery_3452 | Q10YY0 |
| Carbamoyl-phosphate synthase large chain | carB | Q110M4 | Heme oxygenase (Decyclizing) | Tery_0335 | Q119L5 |
| Ferredoxin (2Fe-2S) | Tery_0914 | Q117L1 | Hemolysin-type calcium-binding region | Tery_3467 | Q10YW6 |
| Glutathione S-transferase-like | Tery_4017 | Q10XI9 | L-threonine synthase | Tery_4348 | Q10WN4 |
| Methyl-accepting chemotaxis sensory transducer | Tery_4229 | Q10WZ4 | Methyltransferase type 12 | Tery_1776 | Q114N9 |
| Na-Ca exchanger/integrin-beta4 | Tery_0599 | Q118N1 | Nitrogenase cofactor biosynthesis protein NifB | Tery_4133 | Q10X83 |
| Nucleotidyl transferase | Tery_1143 | Q116R9 | Nitrogenase iron protein | nifH | O34106 |
| Photosystem I protein PsaD | Tery_3791 | Q10Y37 | PBS lyase HEAT-like repeat | Tery_0995 | Q117F1 |
| Phycobilisome linker polypeptide | Tery_0985 | Q117F9 | Phosphonate ABC transporter, periplasmic phosphonate-binding protein | Tery_4993 | Q10V17 |
| Phycobilisome protein | Tery_0996 | Q117F0 | Phycobilisome protein | Tery_0983 | Q117G1 |
| Phycobilisome protein | Tery_0998 | Q117E8 | Putative signal transduction protein with Nacht domain | Tery_2496 | Q111X1 |
| Signal recognition particle protein | ffh | Q111T7 | Ribulose 1,5-bisphosphate carboxylase small subunit | Tery_4408 | Q10WH8 |
| Transcriptional regulator AbrB | Tery_1859 | Q114F9 | Ribulose bisphosphate carboxylase large chain | cbbL | Q10WH8 |
| Uncharacterized protein | Tery_0741 | Q118A2 | Shikimate kinase | aroK | Q115N2 |
| Uncharacterized protein | Tery_0766 | Q117Y1 | Twitching motility protein | Tery_1747 | Q114R7 |
| Uncharacterized protein | Tery_1805 | Q114L2 | Uncharacterized protein | Tery_3106 | Q10ZT5 |
| Uncharacterized protein | Tery_3933 | Q10XR0 | Uncharacterized protein | Tery_3845 | Q10XY7 |
|  |  |  | Uncharacterized protein | Tery_3895 | Q10XU4 |
|  |  |  | WD-40 repeat | Tery_4625 | Q10VX7 |
