# Supplementary material for: Transcriptional and Proteomic Choreography Under Phosphorus Deficiency and Re-supply in the N2 Fixing Cyanobacterium Trichodesmium erythraeum
Source: Front Microbiol. 2019 Mar 5;10:330. doi: 10.3389/fmicb.2019.00330 (PMC6411698; doi:10.3389/fmicb.2019.00330)
Supplement: Supplementary file 3 [file Image_1.pdf]

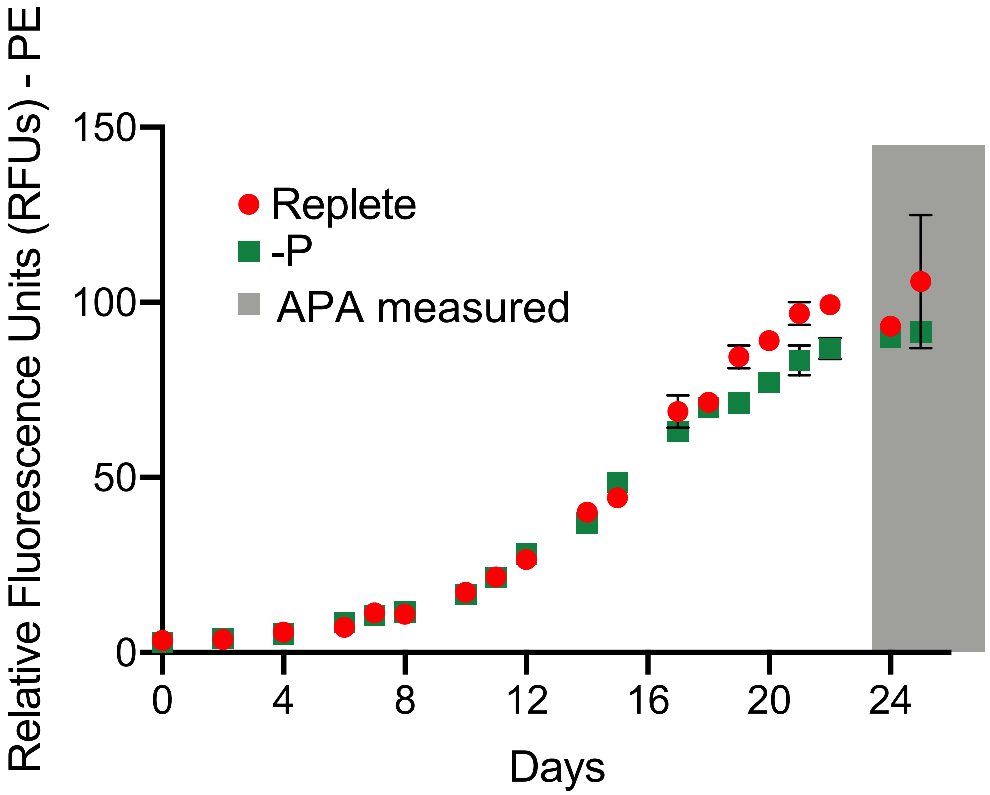

Supplementary Figure 1. Growth curves for phosphorus replete (+P) and phosphorus deficient cultures (−P) assayed with phycoerythrin (PE) fluorescence and plotted in relative fluorescence units. Over the exponential phase (days 6–17), the −P and +P growth rates were 0.19 and 0.2 d^{-1}, respectively. The gray bar indicates when alkaline phosphatase activity (APA) was measured. Error bars represent standard error of the mean (SEM), where n = 3 for +P and n = 6 for −P.
